# Universal ultrasound screening and early treatment of developmental dysplasia of the hip: a critical review

**DOI:** 10.25122/jml-2024-0251

**Published:** 2024-08

**Authors:** Nabil Alassaf

**Affiliations:** 1 Department of Orthopedic Surgery, Hail Health Cluster, Hail, Saudi Arabia

**Keywords:** universal screening, selective screening, hip dysplasia, congenital hip dislocation, pediatric

## Abstract

Developmental dysplasia of the hip (DDH) is the most common musculoskeletal disease in infants, and delayed diagnosis can worsen the prognosis. Clinical evidence increasingly supports universal ultrasound (US) screening over selective US screening. The Graf method remains the most widely accepted US technique. Performing an US screening at one month of age seems appropriate as it allows for some hip maturity and early detection, thereby increasing the chances of a favorable outcome. This paper presents an approach to US findings based on the femoral head coverage method. Considering the long-term cost and psychosocial impact of missed DDH cases, universal ultrasound screening appears to be a cost-effective alternative.

## INTRODUCTION

Developmental dysplasia of the hip (DDH) is a spectrum of pathologies ranging from mild, self-resolving dysplasia to irreducible dislocation. Its etiology is believed to involve genetic and mechanical factors, with key determinants including breech presentation, female gender, family history, and oligohydramnios [1,2]. Traditional swaddling methods, which adduct and extend the hips, increase the risk of hip dislocation, while other risk factors include being a firstborn child, high birth weight, foot deformities, and multiple pregnancies [3,4].

The incidence of DDH varies considerably based on the definition of dysplasia, detection method, and geographic location [5]. In a single hospital in the western province of Saudi Arabia, the incidence rate of DDH was found to be 12 per 1,000 live births when using ultrasound (US) screening following clinical assessment [6]. DDH is believed to be more prevalent among Gulf Cooperation Council nationals [7].

History and clinical examination alone are specific but not sensitive enough to screen DDH [8-10]. Meanwhile, ultrasound is 100% sensitive [11]. Screening for DDH is a form of secondary prevention. Harper *et al*. [12] found that experienced pediatric orthopedic surgeons mislabeled 14% of the dislocated hips as reduced based on physical examination alone. Kyung *et al*. [13] also noted significant inconsistency between clinical and US findings despite examinations by seasoned orthopedic surgeons.

The most commonly prescribed treatment for DDH during the first 6 months of life is the Pavlik harness (PH). The failure rate of PH varies widely, reported to be as low as 1.8% and as high as 29% in one study, where the success rate deteriorated with age [1,14-15]. When splinting fails, or the child presents at more than approximately six months, reduction is usually done in the operating room. The age at the time of surgery is an independent predictor of the need for more invasive procedures [16,17]. Early treatment with an abduction splint leads to better outcomes compared to late presentation after the child begins walking [18].

Mass screening for DDH in infancy has been long recommended and linked to the degree of country development [19]. Despite growing clinical evidence, the topic remains controversial, with significant inter and intra-country differences. People in the high-risk group have a higher proportion of affected individuals, but the low-risk group probably has a larger number of cases. The Geoffrey Rose population-based prevention strategy takes this into account [20]. In other words, most cases will be missed when screening subjects with risk factors using a targeted approach. In comparison, the opposite is true when utilizing universal screening. Rose also quoted the term 'prevention paradox', indicating that a prevention measure that benefits the population has a small value for participating individuals, which lowers the motivation to participate. However, a population-based approach enhances the concept of equality in healthcare delivery [21].

Various imminent entities have released guidelines indicating that there is insufficient evidence to support universal US screening, such as the American Academy of Pediatrics (2000), The Canadian Task Force (2001), the United States Preventive Services Task Force (2006), Pediatric Orthopedic Society of North America (2007), European Society of Pediatric Radiology (2011) and more recently the American Academy of Orthopedic Surgeons (2014) [22-26]. Since the release of such guidelines, however, multiple studies have been published. This review aimed to re-examine and summarize peer-reviewed literature pertinent to the screening and early treatment of DDH.

### Screening programs

To compare the efficacy of universal versus selective US screening for DDH, a MEDLINE search was conducted using the terms 'Screening' and 'DDH'. This comprehensive search was performed across all fields without filters, with the last search conducted in July 2024. Articles were selected based on their titles and abstracts, and references within these articles, as well as papers citing them, were assessed and reviewed.

Health authorities universally expect that routine neonatal physical exams include an assessment for DDH. There are two primary US screening techniques for early detection of DDH: a population-based mass approach, commonly known as universal screening, and a high-risk targeted approach, referred to as selective US screening. Universal ultrasound screening using the Graf method has been performed in at least six European countries, with the initial ultrasound conducted between the first and 90^th^ day of life. Conversely, in Denmark, Greece, Hungary, and France, US screening is performed selectively based on the presence of risk factors and physical exam findings. However, the availability of high-quality clinical assessments may not be consistent [27,28].

Notably, 94% of the members of the Pediatric Orthopedic Society of North America believe that a universal screening program should not be adopted in the United States and that 'high-risk' selective screening is adequate. Interestingly, 13% of respondents reported over-referral of patients for suspected DDH, while the majority noted seeing DDH patients older than 1 year for their initial assessment [29]. A Cochrane review by Shorter *et al*. [30] in 2011 concluded that there is insufficient evidence to make clear recommendations for or against universal US screening [30]. Selective screening can be subjective and varies between clinicians, though it seems more effective in certain parts of the world. For example, in Sweden, the Swedish Pediatric Orthopedic Society has been auditing missed cases through a registry of late-diagnosed hip dislocations since 2000, considering DDH detection after two weeks of age as a late diagnosis. The incidence of late detection went down to 0.1 per 1000 live births from 0.9 per 1,000 live births [31]. Holen *et al*. concluded that universal ultrasound is not necessary in the presence of 'high quality' selective screening, and this was based on a randomized controlled trial (RCT) of 15,529 infants [32]. There was one late detection in the universal screening arm and five late detections in the selective screening group, which did not reach statistical difference [32]. Similarly, an RCT of 11,925 infants by Rosendahl *et al*. found fewer cases of late detection in the universal screening arm, though the difference was not statistically significant [33]. RCTs require clinical equipoise and are likely to show differences with larger samples. However, it remains uncertain if the controlled setting in RCTs is generalizable to broader clinical practice [34].

Clarke *et al*. reported on a selective screening program at a maternity hospital in the UK, where 19% of infants were screened by US based on clinical assessment, resulting in only 4.6% of DDH cases presenting after 3 months [35]. Another South Australian study noted that 2.4% of DDH cases presented after 3 months of age using a selective US screening approach, which included serial clinical assessments for DDH by trained staff until the age of 2.5 years [36]. In a systematic review of observational studies by Kuitunen *et al*., the authors concluded that universal screening has a higher rate of early detections and treated patients, but the incidence of late detection and surgical treatment was not significantly lower than with selective screening [37].

Kamath *et al*. failed to document a significant reduction in DDH late detection in the Greater Glasgow area, Scotland, UK, after the initiation of a selective US screening program [38]. In some health systems where selective US screening is chosen, the referral for hip US is high, probably because of the medico-legal risk [39,40]. An international multi-specialty panel of 24 experts strongly favored universal US screening as early as possible, before the sixth week of age, and agreed that universal US screening is cost-effective and does not lead to over-treatment. These experts reviewed current evidence before voting [2]. Johnson *et al*. [41] demonstrated that about 50% of DDH cases would be missed with a selective ultrasound approach, a finding similar to that of Ziegler *et al*. [10]. Gyurkovits *et al*. noted that about half of DDH patients did not exhibit any physical signs or risk factors other than female gender [42]. Using a universal screening ap-proach, Buonsenso *et al*. [43] found that 19 out of the 48 (40%) pathologic hips had no risk factor or positive clinical findings. Talbot *et al*. [44] noted that 58% of irreducible hips presented late despite a selective screening program. In a meta-analysis by Laborie *et al*. [45], which included 511,403 patients, selective ultrasound screening was inferior to universal ultrasound in early detection of DDH [45]. This was similar to what was reported in an earlier meta-analysis by Jung and Jang [46]. In another review of 12 studies by Poacher *et al*. [47], selective screening did not reduce the rate of surgical intervention in the UK, which is 0.8/1000 live births. Furthermore, this approach has become less effective over the years, according to Sharrock *et al*. [48]. Broadhurst *et al*. studied the rate of late detection of DDH, defined as diagnosis after the age of one year, and found that the rate had not decreased over 35 years of a national selective screening program in the UK, and it increased from about 0.45 to 1.28 per 1,000 live births [49]. This concurred with the conclusions of other reports two decades earlier [50-52].

In Germany, universal ultrasound screening is recommended and provided for free for all newborns. If DDH is suspected, the US is performed during the first week of life; otherwise, it is scheduled between the ages of 4 to 6 weeks. Von Kries *et al*. [53] found that universal screening led to 50% fewer operative procedures in patients who complied with the recommendation compared to those who did not undergo universal US, aligning with earlier findings by Wirth *et al*. [54]. Thallinger *et al*. also noted that the universal screening program in Austria reduced pelvic osteotomies by 46% [55]. Sink *et al*. reviewed 68 skeletally mature patients with symptomatic hip dysplasia requiring corrective osteotomy and found that the current selective ultrasound screening program would miss 85% of those patients [56]. A summary of the evidence is outlined in Table 1.

**Table 1:** Summary of systematic reviews and meta-analyses that compared universal with selective ultrasound screening during the last 20 years

Author, Study	Year	Description	Number of studies/Participants	Conclusion
Laborie *et al*. [45]	2023	SRMA	16/511,403	Late detections are lower with UUS
Cheock *et al*. [57]	2023	SRMA	31	Late detections are lower with UUS
Kuitunen *et al*. [37]	2022	SRMA	76/16,901,079	Higher early detections with UUS
Pandey and Jojari [58]	2021	SR	34	Late detections are lower with UUS
Jung and Jang [46]	2020	SRMA	5/59,492	Late detections are lower with UUS
Shorter *et al*. [30]	2013	SR	2/23,530	Insufficient evidence

SR, systematic review: SRMA, systematic review and meta-analysis; UUS, universal ultrasound screening.

### Ultrasound techniques

Ultrasound remains superior to radiographs in diagnosing DDH in the first few months of life in terms of ionizing radiation exposure and accuracy [59]. The Graf method is considered the reference standard for ultrasound diagnosis of DDH and is performed with the hip flexed in a lateral decubitus position [27]. The alpha and beta angles, which are key to this method, are reliably interpreted by examiners, though the quality of the images can impact their accuracy [60-62]. When combined with certain anthropometric data, automated ultrasound scanning shows promising clinical applications by minimizing the need for manual measurements [63]. Additionally, artificial intelligence-guided portable ultrasound image acquisition and reporting are being investigated to reduce costs and minimize false-positive referrals [64].

The femoral head coverage (FHC) method is another reliable technique for assessing DDH. It classifies hips as normal if the FHC is more than 50%, unstable if 40-49%, subluxated if 30-39%, and dislocated if less than 30% [65-67]. The American Academy of Orthopedic Surgeons defines FHC categories as normal (≥45%), borderline (35-44%), or dysplastic (<35%). Husum *et al*. suggested a pubofemoral distance cut-off value of more than 4.4 mm to indicate dysplasia, with 100% sensitivity and 93% specificity for DDH [68].

### Ultrasound timing

In Austria, universal US screening is performed at least twice: once during the first week of life and again between 6-8 weeks. In the Czech Republic, US is universally performed three times during the first three months [28]. Graf recommends conducting US screening before six weeks of age [69]. The American College of Radiology suggests that hip US should be done after four weeks of age [70], while both the American Institute of Ultrasound in Medicine and the American College of Radiology advise against performing hip US on infants younger than 3 to 4 weeks unless there are clinical signs of hip instability [71].

One main disadvantage of US screening after hospital discharge is that a certain percentage of infants may not be brought in for their US appointment [72]. For busy facilities, an after-hours US screening may reduce the load on the health system and minimize parent absence from work. A delay of treatment up to 6 weeks does not seem harmful [73], though the success rate of the PH decreases if treatment begins after six weeks [45,74,75]. Sanghrajka *et al*. [76] noted that none of the 55 patients who underwent open reduction had started PH treatment before six weeks. Therefore, infants ideally should be seen by a pediatric orthopedic service before 6 weeks of age.

Based on the literature, four weeks of age for infants who can reliably be brought to later US appointments appears most appropriate, allowing for some degree of hip maturity and an early appointment in the pediatric orthopedic clinic. Otherwise, doing US before discharging the infant is a viable alternative [55,77]. There is concern that early US may show many hips as pathologic, but a study of 21,676 newborns who underwent US screening during the first week of life found that only 0.3% of the hips were pathologic [78]. Another study of 28,000 consecutive live births universally screened by US during the first few days of life reported no late DDH presentations within the first 5 years of life [79]. In a review by Sakkers and Pollet, Graf 2a hips have an 89% to 98% chance of spontaneous recovery, and the percentage deteriorates to less than 50% in Graf 4 [80]. When treatment is initiated for unstable hips, US needs to be repeated after two weeks [14], and an X-ray should be requested for treated patients before discharging them from the clinic [81]. A recent report by Hockett *et al*. found that premature birth does not influence hip maturity and thus does not affect US timing [82].

### Early treatment

Monitoring borderline cases without treatment during the first three months of life can be justified. However, the parents need to be aware of the possible need for abduction bracing based on subsequent imaging [83]. In one study, investigators monitored 42 hips with a mean FHC of 39% at one month of age. At the 2-year follow-up, radiographs showed an acetabular index (AI) of 22 degrees, with none of the hips exceeding 30 degrees [84]. In an RCT by Pollet *et al*. [85], 80% of borderline dysplasia (Graf IIb/IIc) diagnosed at age 3-4 months normalized spontaneously. With active surveillance, the number of treated patients could be reduced, though there are concerns about losing follow-up, and some parents may not perceive the delay in treatment favorably. Therefore, treating these cases with more convenient splints like a Frejka pillow might be safer, as suggested by Blom *et al*. [86]. Zídka and Dzupa used a Frejka pillow for milder cases and the PH for the more unstable cases, noting higher non-compliance with the PH [15]. The decision to continue treatment should not be based solely on ultrasound; radiographic indices, particularly the acetabular index, must also normalize. [87-89]. Ultrasound results are best interpreted in the context of risk factors [90].

The author’s preferred treatment approach is to apply a PH if FHC is less than 30% and use a Frejka pillow if FHC is 30% to 39%. For an FHC between 40% and 49%, treatment with a Frejka pillow is started if the child has significant risk factors like breech presentation, positive family history, oligohydramnios, and or multiple gestations. Otherwise, the child is observed with no treatment, reassessed after 3 months of age, and managed accordingly. An FHC of 50% or more does not require further management and follow-up. Abduction splinting is continued until indices normalize on plain radiographs.

### The burden of universal ultrasound screening

Over-treatment, if indeed an issue, is not the direct result of universal ultrasound screening. The clinician's understanding of the ultrasonic findings influences the decision to treat. Treatment of borderline cases, often referred to as 'stable hips', can be deferred until 3 to 4 months of age, when a radiograph can be obtained to confirm if the infant has an immature or dysplastic hip. The Cochrane review by Shorter *et al*. concluded that there is inconsistent evidence about whether universal ultrasound increases treatment [30].

Regarding the risk of avascular necrosis (AVN), Gahleitner *et al*. reviewed 60 patients over an average of 20.5 years following PH treatment, initiated and abandoned based on US findings. No AVN cases were reported, and only two hips showed residual dysplasia [91]. Rosendahl reported using Frejka pillow in approximately 1,200 infants with no resulting AVN [92].

Graf noted that the cost of screening and subsequent treatment is 33% less than the cost of treatment before the universal US screening program [69]. It is helpful to understand that reducing late detections means less prolonged spica casting, open reductions, pelvic and femoral osteotomies, revision open reductions, AVN, stiffness, leg length discrepancies, and early hip replacements. Therefore, a precise cost-benefit analysis is challenging, and the argument is more ethical. Studies often overlook the psychosocial impact and long-term costs.

Thaler *et al*. compared two five-year periods in the province of Tyrol, Austria. During the first period (1978 to 1982), screening was solely based on clinical examination before the widespread use of the Graf ultrasound method. In the second period (1993 to 1997), universal ultrasound screening was established. They noted an overall cost increase of €57,000 per annum in the second period but a seventy-six percent reduction in treatment interventions [93]. Clegg *et al*. in Coventry, UK, found that the overall cost of universal screening is comparable to that of surgical treatment [92]. This conclusion is similar to that of Rosendahl *et al*. in Bergen, Norway, who also noted that the cost of screening decreased over time following the implementation of universal US screening [94]. Woodacre *et al*. studied a regional selective ultrasound screening program with referrals to a hip dysplasia clinic in Exeter, UK. They found that 19% of cases were missed by the program and presented later than three months. The cost of US screening was £56/child, while the average cost of surgery was £4,352/child [95].

## CONCLUSION

Universal US screening using the Graf method is justified and facilitates early detection and treatment, hence providing a better prognosis with no or minimal long-term added cost. Over-treatment can be avoided by a better understanding of US findings and by using treatment protocols that allow observation of milder forms of ultrasonographic hip dysplasia.
